# Influenza Transmission Dynamics in Urban Households, Managua, Nicaragua, 2012–2014

**DOI:** 10.3201/eid2410.161258

**Published:** 2018-10

**Authors:** Aubree Gordon, Tim K. Tsang, Benjamin J. Cowling, Guillermina Kuan, Sergio Ojeda, Nery Sanchez, Lionel Gresh, Roger Lopez, Angel Balmaseda, Eva Harris

**Affiliations:** University of Michigan, Ann Arbor, Michigan, USA (A. Gordon);; The University of Hong Kong, Hong Kong, China (T.K. Tsang, B.J. Cowling);; Ministry of Health, Managua, Nicaragua (G. Kuan, R. Lopez, A. Balmaseda);; Sustainable Sciences Institute, Managua (S. Ojeda, N. Sanchez, L. Gresh);; University of California, Berkeley, California, USA (E. Harris)

**Keywords:** household, influenza, Nicaragua, Managua, serial interval, transmission, viruses, respiratory infections, influenza A, influenza B, H1N1, H3N2, risk for infection, household transmission, oseltamivir, age, household contacts, vaccination

## Abstract

During August 2012–November 2014, we conducted a case ascertainment study to investigate household transmission of influenza virus in Managua, Nicaragua. We collected up to 5 respiratory swab samples from each of 536 household contacts of 133 influenza virus–infected persons and assessed for evidence of influenza virus transmission. The overall risk for influenza virus infection of household contacts was 15.7% (95% CI 12.7%–19.0%). Oseltamivir treatment of index patients did not appear to reduce household transmission. The mean serial interval for within-household transmission was 3.1 (95% CI 1.6–8.4) days. We found the transmissibility of influenza B virus to be higher than that of influenza A virus among children. Compared with households with <4 household contacts, those with >4 household contacts appeared to have a reduced risk for infection. Further research is needed to model household influenza virus transmission and design interventions for these settings.

## Abstract 2

In this low-income country setting, ≈16% of household contacts acquired infections from index patients, despite high oseltamivir use.

Influenza virus is a respiratory pathogen of major medical and public health concern, causing an estimated 3–5 million cases of severe illness and 250,000–500,000 deaths annually worldwide (*1*). Households provide a convenient and valuable setting for studying the transmission of influenza virus (*2*–*5*). Several studies in high-income country settings suggest that the rate of influenza virus transmission in the household is several-fold higher than that in the community (*3*,*4*). In a study conducted in Vietnam, 26% of influenza virus infections were acquired in the household (*6*). The influence of household transmission on influenza epidemics has led to an increased interest in household-based interventions (*7*–*11*). However, in low-income and low-middle–income countries, where nearly half the world’s population lives, household influenza transmission has not been well studied. Moreover, estimates of the serial interval (i.e., time between index case and symptom onset in secondary infection) are limited to pandemic influenza in nonhousehold settings (*6*,*12*–*15*). Therefore, a more thorough investigation of household transmission is essential for the development of effective household-based interventions for the control of pandemic and interpandemic influenza in these settings.

Demographic factors that have been found to influence influenza transmission include the size of the household, age of the index patient, and age of contacts (*4*,*6*,*16*–*19*). Both household size and population demographics differ dramatically between industrialized and developing country settings. In Nicaragua, persons from several generations often live in the same household, leading to large household sizes by high-income country standards. In addition, in 2014, ≈32% of the population of Nicaragua was <15 years of age, whereas in other countries where influenza household transmission studies have been conducted, 12%–23% of the population was estimated to be in this age range (*5*,*6*,*20*–*22*).

To investigate influenza transmission in households, we performed a case ascertainment study of influenza in urban households in Managua, Nicaragua. We used an individual-based hazard model to characterize transmission dynamics within households and estimate factors affecting influenza transmission.

## Materials and Methods

### Study Subjects

Index influenza cases were identified at the Health Center Sócrates Flores Vivas, a primary care facility in Managua, Nicaragua, run by the Ministry of Health of Nicaragua. Index patients were eligible for enrollment if a) they had influenza-like illness, defined as fever or feverishness with cough, sore throat, or runny nose; 2) their symptom onset, defined as the earliest day with influenza-like illness, was within the previous 48 hours; 3) they were positive for influenza by rapid antigen test or reverse transcription PCR (RT-PCR); 4) no household members had had symptoms of influenza-like illness in the previous 2 weeks; and 5) they lived with >1 additional person. After index case enrollment, we conducted a household visit to enroll patient household contacts, collect initial respiratory and blood samples, and administer questionnaires to the household and individual household members. We defined a household as a group of persons living together who shared a kitchen and >1 meal a day. We visited households 4 additional times (every 2–3 days) to collect respiratory samples and daily symptom information, and we collected the final blood sample 30–45 days after index case enrollment.

This study was approved by the institutional review boards at the Ministry of Health of Nicaragua, the University of Michigan (Ann Arbor, Michigan, USA), and the University of California, Berkeley (Berkeley, California, USA). Consent to participate was obtained from all adult participants, and parental permission was obtained for all children. Assent was obtained for children >6 years of age.

### Laboratory Methods

We stored nasal and throat swab samples at 4°C–8°C and transported them to the National Virology Laboratory (Managua, Nicaragua) within 12 hours. We tested all samples for influenza on an ABI 7500 Fast PCR platform (Applied Biosystems, Foster City, CA, USA) following validated protocols from the US Centers for Disease Control and Prevention (Atlanta, GA, USA).

### Statistical Analysis

We characterized influenza transmission dynamics within households and the effects of factors affecting transmission using an individual-based hazard model (*5*,*17*). In the model, the risk for RT-PCR–confirmed infection among household contacts depended on the time from symptom onset of other infected persons in the household. The hazard (*λ*) of infection of person *j* at time *t* from an infected household member *i*, with symptom onset *t_i_* is *λ_i→j_* (*t*) = λ*_n_* × *S_j_*, where λ*_n_* is the baseline hazard of household transmission and *S_j_* is the factors affecting transmissibility.

The distribution of the serial interval was a discretized Weibull distribution (*23*), with probability mass function

 ,where *t* is the number of days after symptom onset of the index patient, *α* is the shape, and *γ* is the scale parameter for the distribution. The model used to estimate the distribution included infection of household contacts by persons inside the household (index cases [i.e., secondary infections] and other infected household members [i.e., tertiary infections]) and outside the household (i.e., community infections). The hazard of infection from the community was assumed to be constant over the duration of the follow-up: λ*_j,c_* (*t*) = ψ, where ψ is the baseline community risk (*5*). Therefore, the hazard of infection for a person *j* at day *t* is λ*_j_* (*t*) = λ*_j,c_* (*t*) + ∑*_i_* λ*_i→j_* (*t*), and the summation involves the infected household contacts of person *j* only.

In the transmission model, age group (<18 years vs. >18 years) and vaccination status of the household contacts were factors that might affect the susceptibility of household contacts to infection (*5*,*17*). In addition, we accounted for the possible differences in transmission related to the different influenza virus types [influenza A(H1N1), influenza A(H3N2), and influenza B]; treatment (with vs. without oseltamivir) of index case; index patient age group (children ≤5 years of age vs. older children and adults); and household sizes, hereafter denoted as the preliminary analysis. In addition to these factors, we further explored the differences in the relative susceptibilities of children and adults to influenza types A and B, which was represented by an interaction of age and influenza types in the model. We considered this analysis the main analysis (Technical Appendix).

We fitted this model into a Bayesian framework, constructed a Markov chain Monte Carlo algorithm, and estimated parameters (*24*). We used conditional likelihood in the statistical model to account for the study design feature that no household contacts had symptom onset at or before the day of index case enrollment (Technical Appendix). To evaluate model adequacy, we conducted a simulation study with the model to compare the estimated and observed risks of groups with different characteristics (Technical Appendix). We performed statistical analyses with R version 3.1.1 (https://cran.r-project.org/) and MATLAB version 7.8.0 (https://www.mathworks.com/products/matlab.html).

## Results

**Figure 1:**
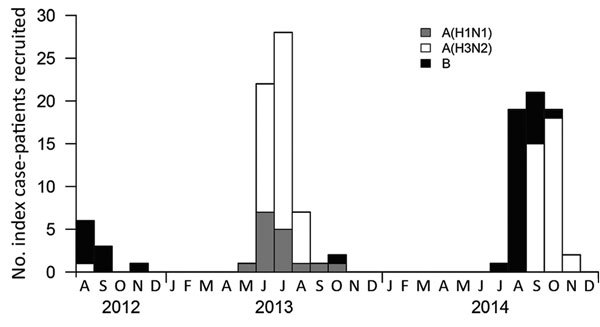
Timeline of enrollment of index cases of PCR-confirmed monoinfections of seasonal influenza A(H1N1) virus, influenza A(H3N2) virus, or influenza B virus, Managua, Nicaragua, August 2012–November 2014. Only the index cases included in the final analysis are shown.

During August 2012–November 2014, a total of 168 potential index patients consented to participate in the study. We excluded 6 households with multiple index cases, 5 households with influenza virus infections of mixed subtypes, and 24 households with index cases that were initially positive for influenza by rapid antigen test but not confirmed positive by RT-PCR. In total, 133 households with index cases of influenza virus infection confirmed by RT-PCR were included in our analysis (Figure 1). At the initial visit, 541 household contacts were present, and 536 (99%) were enrolled. A total of 2,285 respiratory samples were collected from household contacts (mean 4.3 respiratory samples/contact). Of the 356 household contacts, 84 (15.7%, 95% CI 12.7%–19.0%) had RT-PCR–confirmed influenza virus infections (Table 1). Of these, 21 (25%) did not exhibit symptoms. Influenza transmission was observed in 52 (39%) households. Of the households with influenza transmission, 34 had 1 contact with an RT-PCR–confirmed influenza virus infection, 10 had 2 contacts, and 8 had >3 contacts. Most index cases (76%) were managed with oseltamivir. The average age of index patients was 6.6 (range 0–45) years, and the average age of household contacts was 24.2 (range 0–87) years. Mean household size was 5.0 (range 2–17) members. Among household contacts, the overall observed risk for influenza A(H1N1) virus infection was 13.4% (9/67, 95% CI 6.3%–24.0%), influenza A(H3N2) virus 14.3% (46/322, 95% CI 10.7%–18.6%), and influenza B virus 19.7% (29/147, 95% CI 13.6%–27.1%).

**Table 1:** Characteristics of influenza virus–infected index cases-patients and household contacts, Managua, Nicaragua, August 2012–November 2014*

Characteristic	Influenza type, no./total (%)			Total, no. (%)
	A(H1N1)	A(H3N2)	B	
Index patients	16	80	37	133
Age, y				
<5	10 (63)	52 (65)	15 (41)	77 (58)
6−18	5 (31)	23 (29)	21 (57)	49 (37)
>18	1 (6)	5 (6)	1 (3)	7 (5)
Sex				
F	6 (38)	36 (45)	17 (46)	59 (44)
M	10 (63)	44 (55)	20 (54)	74 (56)
Prior vaccination				
Yes	1 (6)	2 (3)	3 (8)	6 (5)
No	15 (94)	78 (98)	34 (92)	127 (95)
Oseltamivir treatment				
Yes	16 (100)	53 (66)	32 (86)	101 (76)
No	0	27 (34)	5 (14)	32 (24)
No. household contacts				
1–3	6 (38)	44 (55)	23 (62)	73 (55)
4–5	5 (31)	19 (24)	6 (16)	30 (23)
>5	5 (31)	17 (21)	8 (22)	30 (23)
No. secondary cases in household				
0	10 (63)	54 (68)	17 (46)	81 (61)
1	4 (25)	16 (20)	14 (38)	34 (26)
2	1 (6)	4 (5)	5 (14)	10 (8)
>2	1 (6)	6 (8)	1 (3)	8 (6)
Household contacts	67	322	147	536
Age, y				
<18	30 (45)	147 (46)	64 (44)	241 (45)
>18	37 (55)	175 (54)	83 (56)	295 (55)
Sex				
F	46 (69)	202 (63)	94 (64)	342 (64)
M	21 (31)	120 (37)	53 (36)	194 (36)
Prior vaccination				
Yes	3 (4)	9 (3)	20 (14)	32 (6)
No	64 (96)	313 (97)	127 (86)	504 (94)
With confirmed infection				
Overall	9/67 (13)	46/322 (14)	29/147 (20)	84/536 (16)
<18 y†	3/30 (10)	33/147 (22)	21/64 (33)	57/241 (24)
>18 y†	6/37 (16)	13/175 (7)	8/83 (10)	27/295 (9)
No. confirmed infections without reported symptoms				
Overall	2/9 (22)	15/46 (33)	4/29 (14)	21/84 (25)
<18 y†	2/3 (67)	11/33 (33)	3/21 (14)	16/57 (28)
>18 y†	0/6	4/13 (31)	1/8 (13)	5/27 (19)

*Not all percentages add up to 100% because of rounding. †The denominator is the number of infected household contacts in the corresponding age group.

In the preliminary analysis, we adjusted for age group of household contacts, vaccination history, index patient age group, index case treatment status, and household contact number but did not include the interaction of age group of household contacts and influenza type and subtype. In this analysis, we found that household contacts of index patients with RT-PCR–confirmed influenza B virus infections were more likely to get infected than those of index patients with influenza A(H3N2) virus infections (relative infectivity 1.71, 95% CI 1.08–2.80) or influenza A(H1N1) virus infections (relative infectivity 1.56, 95% CI 0.75–3.43).

In the main model, we included the interaction of age group of household contacts and influenza type, which accounted for the difference in relative susceptibility between children and adults for influenza A and influenza B. Using this model, we found no differences in risk for infection between influenza A(H3N2) virus and influenza A(H1N1) virus or influenza B virus (Table 2). This finding suggested that the observed increased susceptibility to infection with influenza B virus could be explained by an interaction between age of household contacts and influenza type; in other words, child contacts were more susceptible to infection with influenza B virus than influenza A virus, and among adult contacts, the risk for infection with influenza A virus was similar to the risk for infection with influenza B virus.

**Table 2:** Factors affecting influenza transmission in urban households, Managua, Nicaragua, August 2012–November 2014

Characteristics	Risk ratio (95% CI)
Influenza type	
A(H3N2)	Referent
A(H1N1)	1.18 (0.5–2.42)
B	0.96 (0.4–2.15)
Age of household contact, y	
>18	Referent
<18 for influenza A	2.26 (1.38–3.88)
<18 for influenza B	4.47 (2.05–11.02)
Prior vaccination of household contact	
No	Referent
Yes	0.46 (0.11–1.32)
Age of index patient, y	
<5	Referent
>5	1.55 (0.98–2.45)
Oseltamivir treatment of index case	
No	Referent
Yes	0.69 (0.42–1.12)
No. household contacts	
1–3	Referent
4–5	0.60 (0.30–1.10)
>5	0.69 (0.37–1.18)

We estimated that child household contacts (<18 years of age) were more susceptible to RT-PCR–confirmed influenza A virus infection than adult contacts (>18 years of age) (relative susceptibility 2.26, 95% CI 1.38–3.88), and child contacts were more susceptible to RT-PCR–confirmed influenza B virus infection than adult contacts (relative susceptibility 4.47, 95% CI 2.05–11.02). Because there were only 7 adult index patients (Table 1), we could not explore potential differences in infectivity of child versus adult cases. However, we did explore relative infectivity of younger children (<5 years of age) versus older children and adults; the estimated relative infectivity of younger children was 1.55 (95% CI 0.98–2.45) (Table 2).

We found no statistically significant association between oseltamivir treatment of index patients and risk for infection among household contacts (risk ratio 0.69, 95% CI 0.42–1.12). We estimated vaccine effectiveness among vaccinated household contacts as 54% (95% CI −32% to 89%). Household contacts of index patients having >4 household contacts had ≈30%–40% lower risk for infection than those of index patients having <4 household members, but this difference was not statistically significant (Table 2; Technical Appendix Table). We estimated that the mean serial interval for within-household transmission was 3.1 (95% CI 1.6–8.4) days (SD 2.0 [95% CI 0.4–10.8] days).

**Figure 2:**
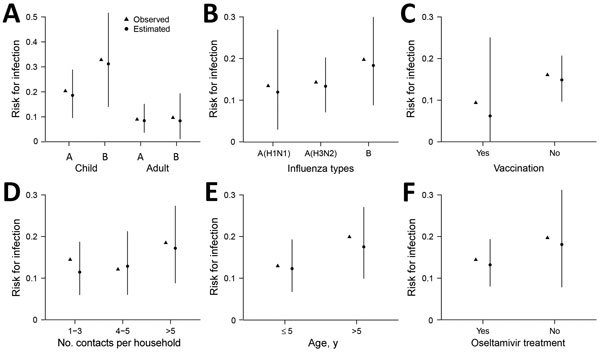
Observed and estimated risks for influenza virus infection of household contacts of index patients with reverse transcription PCR–confirmed influenza virus infections, by characteristic, Managua, Nicaragua, August 2012–November 2014. We estimated risk for infection by performing simulations using a multivariate model fitted to the collected data. Estimates represent 10,000 simulated epidemics in households with a structure that matched exactly that of the observed household. Points indicate medians, and bars represent the 2.5%–97.5% ranges of those 10,000 simulations. Risks for infection are shown for A) child and adult household contacts with influenza A virus infection or influenza B virus infection; B) virus type and subtype; C) vaccinated and nonvaccinated household contacts; D) household contact number; E) age group; and F) household contacts of index patients who were and were not treated with oseltamivir.

We performed simulation studies to assess the adequacy of our model (Figure 2). The median estimated risks for infection among groups from the 10,000 simulated household epidemics were close to the risks observed, suggesting our model provided a reasonable fit of the data.

## Discussion

We describe the results from a case ascertainment study of influenza transmission in urban households of Nicaragua. In this setting, we found the mean serial interval for within-household influenza transmission to be 3.1 days. We further observed an overall risk for RT-PCR–confirmed influenza virus infection of ≈16% among household contacts of index patients with RT-PCR–confirmed influenza virus infections, despite high oseltamivir treatment of index patients (76%).

We found evidence that influenza B virus was more transmissible than influenza A virus, which was explainable by higher transmissibility of this virus among children (Table 2). As expected, children were more susceptible to influenza A and influenza B than adults in our study, presumably because of lower levels of preexisting immunity and different contact patterns (*25*). This finding is consistent with those of other studies (*4*,*6*,*16*,*17*). However, a large randomized controlled trial in households in Thailand did not observe significant differences between children and adults in risk for influenza virus infection (*10*).

We did not detect a significant effect for oseltamivir treatment of index patients on influenza transmissibility. This observation is consistent with several other household transmission studies that have found that oseltamivir treatment decreases the infectious period but does not have a statistically significant effect on the secondary attack rate of laboratory-confirmed influenza (*19*,*26*,*27*). However, other studies have shown a reduction in household transmission from index patients treated with oseltamivir (*28*,*29*). In a review, about half of household transmission studies reported a significant association between index case oseltamivir treatment and reduction in transmission in households, suggesting this issue is still unresolved (*30*). On the other hand, our study might be underpowered to detect this association, considering that 76% of the index patients were treated with oseltamivir.

We did not observe that vaccination had a significant effect on influenza transmission in the household. However, the proportion of contacts vaccinated in this study was low (5%), and thus, the study was underpowered to detect vaccine effectiveness in this population.

We did not find a statistically significant association between risk of acquiring an infection and number of household contacts, although the point estimate suggests that the risk for infection among household contacts of index patients with >4 household contacts was 30%–40% lower than those of index patients with <4 household contacts. This association has also been reported in other studies. The absence of this association in a study might indicate insufficient sample size (*4*–*6*).

We estimated the mean serial interval for influenza in households in Nicaragua to be 3.1 days. This estimate is similar to those found in other settings, such as Hong Kong, where the mean serial interval estimate for influenza A was 3.2–3.6 days (*16*,*21*,*22*); Thailand, where the estimate was 3.3–3.7 days, depending on the type and subtype of influenza (*31*); and Michigan, where the mean serial interval reported was 3.2 days (*32*). In a review of influenza A(H1N1)pdm09 virus transmission, the mean serial interval was estimated to be 2.6 days (*33*).

A major strength of our study was the collection of up to 5 respiratory samples from each household contact, regardless of whether they had symptoms, for 9–12 days after index case identification. However, our study has several limitations, the most notable being that we enrolled index influenza cases only among persons seeking medical care. This aspect of the study design could have biased the study toward sicker than average index patients, which could have inflated our influenza transmission estimate. Also, because adults tend to seek treatment later in their illnesses than children and enrollment was limited to index patients who sought treatment <2 days after symptom onset, our study was overrepresented by index cases in children. This enrollment criterion could have also led to an increase in the intensity of transmission and shortened the observed serial interval of transmission (*4*). Last, not enough adult index cases were enrolled to examine whether child index cases might be more infectious than adult index cases.

In summary, in this household transmission study of influenza in Managua, Nicaragua, we observed a high secondary attack rate of influenza and a serial interval within the range of those observed in high-income country settings. Our findings extend the relatively limited knowledge available regarding influenza transmission in low-middle–income countries. Further research is needed to investigate how household conditions affect influenza transmission and to design household-based interventions in these settings.

Technical Appendix Description of model used to estimate risk for infection, risk for infection by number of household contacts and age, and estimates of posterior distributions of model parameters in study of influenza transmission within households, Managua, Nicaragua, August 2012–November 2014.
