# Supplementary material for: Influenza Transmission Dynamics in Urban Households, Managua, Nicaragua, 2012–2014
Source: Emerg Infect Dis. 2018 Oct;24(10):1882–8. doi: 10.3201/eid2410.161258 (PMC6154158; doi:10.3201/eid2410.161258)
Supplement: Technical Appendix — Description of model used to estimate risk for infection, risk for infection by number of household contacts and age, and estimates of posterior distributions of model parameters in study of influenza transmission within households, Managua, Nicaragua, August 2012–November 2014. [file 16-1258-Techapp-s1.pdf]

Article DOI: https://doi.org/10.3201/eid2410.161258

Influenza Transmission Dynamics in Urban Households, Managua, Nicaragua, 2012–2014

Technical Appendix

Household Transmission Model

We used an individual-based household transmission model to explore the factors affectivity, susceptibility, and infectivity (*1*,*2*). For every person, we observed vectors y_i, d_i, a_i, vac_i, c_i, ose_i, n_i, and sub_i, where y_i is the indictor variable of PCR-confirmed infection, d_i is the symptom onset time, a_i is age (0 for ≤18 years and 1 for >18 years) of household contact, vac_i is the vaccination status (0 for no and 1 for yes), c_i is age (0 for ≤5 years and 1 for >5 years) of index case-patient, ose_i is oseltamivir treatment (0 for no and 1 for yes) of index case-patient, n_i is the number of household members, and sub_i is the influenza subtype [1 for A(H1N1), 2 for A(H3N2), and 3 for B] for person *i*.

Serial Interval

We used the same definition as Cauchemez et al. and Tsang et al. for serial interval (*1*,*2*). We assumed the distribution of the serial interval followed the discretized Weibull distribution, with probability mass function $f_i(t) = \exp\left(-\left(\frac{t}{\gamma}\right)^{\alpha}\right) - \exp\left(-\left(\frac{t+1}{\gamma}\right)^{\alpha}\right), t > t_i$, where *t* is the number of days since symptom onset in the index case-patient.

Person-to-Person Hazard of Infection within Household

We assumed age and vaccination status of household contacts; influenza subtype, age, and oseltamivir treatment of corresponding index case-patient; and number of household members were associated with the hazard of infection and that there was an interaction between age and influenza subtype. Hence, the hazard of infection (λ) of person *j* at time *t* from infected household member *i*, with symptom onset time t_i is $\lambda_{i \to j}(t) = \lambda_n \times \exp\,[\beta_1 I(n_j \geq 4 \& n_j \leq 5) + \beta_2 I(n_j > 5) + \beta_3 I(a_j = 0 \& sub_j = 1 \text{ or } 2) + \beta_4 I(a_j = 0 \& sub_j = 3) + \beta_5 I(vac_j = 1) + \beta_6 I(sub_j = 1) + \beta_7 I(sub_j =$

3) + $\beta_8 I(c_j = 1) + \beta_9 I(ose_j = 1)$]. In this equation, λ_h is the baseline hazard of household transmission. β_1 and β_2 quantifies the relative hazard of infection for household contacts with 4–5 and >5 household members, respectively, compared with those with <4 household members. β_3 and β_4 quantifies the relative hazard of infection for household contacts in the first age group with value 0 (age ≤18 years) compared with those with value 1 (age >18 years) with influenza A virus and influenza B virus, respectively, from the index case-patient. β_6 quantifies the relative hazard of infection for household contacts with vaccination compared with those without vaccination. β_7 and β_8 quantifies the relative hazard of infection for household contacts with influenza A(H1N1) virus and influenza B virus, respectively, compared with influenza A(H3N2) virus from the index case-patient. β_9 quantifies the relative hazard of acquiring infection from the index case-patient for household contacts with oseltamivir treatment versus those without.

Hazard of Infection from Community

Persons might also be infected outside the household (in the community). We assumed the hazard of infection from the community was constant during the duration of the follow-up. Hence the hazard of infection from the community for person j at time t is $\lambda_{j,c}(t) = \psi$, where ψ is the baseline community risk. Although this assumption might be invalid, almost all of the secondary cases in households in household transmission studies have been infected from the index case-patient, as indicated by sequencing analyses (*3*). Hence, the results were insensitive to this assumption.

Total Hazard of Infection

The total hazard of infection for a person j at time t is $\lambda_j(t) = \lambda_{j,c}(t) + \sum_i \lambda_{i\to j}(t)$. The summation is over the infected household members of person j only.

Inference

Likelihood

One particular feature of the study design was that there were no household members with symptom onset at or before the recruitment day. We used a conditional likelihood function to account for this feature. z_{i1} was the start and z_{i2} the end of the follow-up period of person i. On the basis of the transmission model, the probability that a person i was infected, confirmed by

PCR, with infection time t_i is P(y_i = 1, t_i) = [1 – exp (–λ_i (t_i))] × [exp $\left(-\sum_{d=z_{i1}}^{t_i-1}\lambda_i(d)\right)$]. For uninfected case-patients, we denote t_i = z_{i2} +1. The probability that a person *i* does not get infected within the follow-up period is $P(y_i = 0) = \exp\left(-\sum_{d=z_{i1}}^{t_i-1}\lambda_i(d)\right)$. Hence, the log-likelihood function L is $\sum_{i:y_i=1}\log(1-\exp(-\lambda_i(t_i)) - \sum_i\sum_{d=z_{i1}}^{t_i-1}\lambda_i(d)$. Index case-patients do not contribute to the likelihood, and hence, the summation is only on household contacts. For example, for an index case-patient with symptom onset on day 0 and recruitment on day 2, z_{i1} was set to be day 3 for every household contact because household contacts could not have symptom onset before or at day 2 due to the study design. Households containing household contacts with symptom onset at recruitment were excluded in the analyses because this condition violated the inclusion criteria.

Prior

To ensure convergence and efficiency of the Markov chain Monte Carlo estimation, we used prior information that <20% of within-household transmission occurs 14 days after index case-patient symptom onset (*4*). In each update, we rejected the proposed estimates of the parameters of the infectivity profile if $F_{\text{infectivity profile}}(14) \leq 0.8$. For other parameters in the model, we use noninformative prior. For parameters that could only be positive (e.g., parameters for Weibull distribution), we used Uniform(0,10). For other parameters, we used Uniform(−10,10).

Algorithm

Estimation of parameters was performed in a Bayesian framework. The joint posterior distributions of the parameters were explored by Markov chain Monte Carlo. We updated the parameters by using random walk Metropolis-Hastings algorithm. The algorithm ran for 15,000 iterations after a burn-in of 5,000 iterations. Converge was visually assessed.

Model Validation

To validate our model, we simulated 10,000 epidemics in households with a structure that matched that exactly of the observed households and with parameters randomly drawn from their posterior distribution (Technical Appendix Figure). Then, we compared the observed risk for infection with the estimated risk for infection from the model by groups with different characteristics (main text Figure 2). The 2.5%–97.5% range of the 10,000 simulated epidemics

covered the observed risk for infection, and the median of those 10,000 simulated epidemics was close to the observed risk for all groups, suggesting that our model provided a reasonable fit of the data.

References

1. Cauchemez S, Donnelly CA, Reed C, Ghani AC, Fraser C, Kent CK, et al. Household transmission of 2009 pandemic influenza A (H1N1) virus in the United States. N Engl J Med. 2009;361:2619–27. PubMed http://dx.doi.org/10.1056/NEJMoa0905498

2. Tsang TK, Cauchemez S, Perera RA, Freeman G, Fang VJ, Ip DK, et al. Association between antibody titers and protection against influenza virus infection within households. J Infect Dis. 2014;210:684–92. PubMed http://dx.doi.org/10.1093/infdis/jiu186

3. Poon LL, Chan KH, Chu DK, Fung CC, Cheng CK, Ip DK, et al. Viral genetic sequence variations in pandemic H1N1/2009 and seasonal H3N2 influenza viruses within an individual, a household and a community. J Clin Virol. 2011;52:146–50. PubMed http://dx.doi.org/10.1016/j.jcv.2011.06.022

4. Carrat F, Vergu E, Ferguson NM, Lemaitre M, Cauchemez S, Leach S, et al. Time lines of infection and disease in human influenza: a review of volunteer challenge studies. Am J Epidemiol. 2008;167:775–85. PubMed http://dx.doi.org/10.1093/aje/kwm375

Technical Appendix Table. Risk for infection of household contacts and age of contacts, by number of household contacts of index case-patient, Managua, Nicaragua, August 2012–November 2014

No. household contacts	Risk for infection, n/total (%)	Mean age, y	Median age, y
1	2/12 (16.7)	29.5	32.6
2	8/58 (13.8)	25.5	28
3	14/96 (14.6)	27	29.2
4	7/72 (9.7)	21.5	22.8
5	9/60 (15)	20	26
6	16/72 (22.2)	20	21.6
7	5/56 (8.9)	15.5	20.4
8	6/24 (25)	17.5	21.8
9	0/9 (0)	12	16.2
>9	17/77 (22.1)	16	20.9
1–3	24/166 (14.5)	27	29
4–5	16/132 (12.1)	21	24.2
>5	44/238 (18.5)	16	20.9

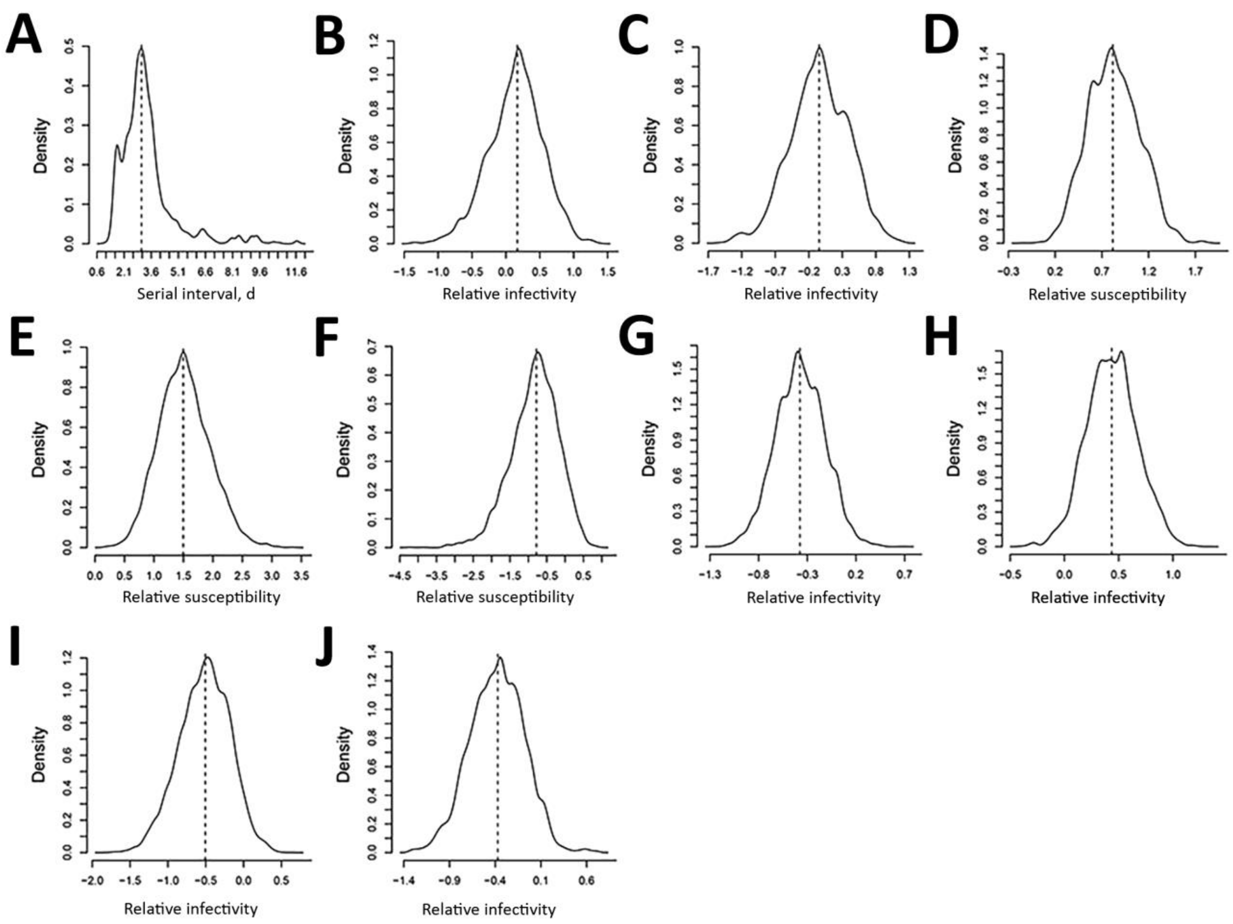

Technical Appendix Figure. Estimated posterior distribution of model parameters. Estimates of effect of factors are in log scale. Dotted vertical line represents posterior estimates. A) Serial interval. B) Relative infectivity between index cases with influenza A(H1N1) and with influenza A(H3N2). C) Relative infectivity between index cases with influenza B and with influenza A(H3N2). D) Relative susceptibility between children and adults for influenza A. E) Relative susceptibility between children and adults for influenza B. F) Relative susceptibility between vaccinated and unvaccinated household contacts. G) Relative infectively between index cases with and without oseltamivir treatment. H) Relative infectivity between index cases of age ≤5 years and age >5 years. I) Relative infectivity between index cases with 3–4 and 1–3 household contacts. J) Relative infectivity between index cases with ≥5 and 1–3 household contacts.
